# Movement, mood and cognition: Preliminary insight into the effects of electroconvulsive therapy in depression through a data-driven resting-state connectivity analysis

**DOI:** 10.1192/j.eurpsy.2021.359

**Published:** 2021-08-13

**Authors:** J.-B. Belge, P. Mulders, J. Van Oort, L. Van Diermen, P. De Timary, E. Constant, P. Sienaert, D. Schrijvers, B. Sabbe, P. Van Eijndhoven

**Affiliations:** 1 Adult Psychiatry, Antwerp University, Antwerp, Belgium; 2 Psychiatry, Radboud University, Nijmegen, Netherlands; 3 Psychiatry, Université Catholique de Louvain, Brussels, Belgium; 4 Psychiatry, UPC KU Leuven, Kortenberg, Belgium

**Keywords:** Neuroimaging, Electroconvulsive therapy, Independent Component Analysis, psychomotor symptoms

## Abstract

**Introduction:**

ECT is an effective treatment for depression. Beyond its therapeutic effect on mood it has a unique impact on psychomotor and cognitive symptoms.Its mechanism of action remains still unclear. To investigate this, we set out to study the brain’s response to ECT from a large-scale brain-network perspective.

**Objectives:**

The aim of this study was to investigate changes in resting-state functional connectivity following ECT at the whole brain, between-network and within-network level, in patients with a depressive episode.

**Methods:**

Resting-state FMRI data were collected from 17 patients with depression before and after an ECT course. Using a group independent component analysis approach, we focused on four networks that are known to be affected in depression: the salience network (SN), default mode network (DMN), cognitive executive network (CEN) and a subcortical network (SCN). Clinical measures including mood, cognition and psychomotor symptoms were assessed.

**Results:**

ECT increased connectivity of the left CEN with the left angular gyrus and left middle frontal gyrus. An increase in left CEN within network connectivity was observed. Both the right CEN and the SCN showed increased connectivity with the precuneus. Furthermore, the anterior DMN showed increased connectivity with the left amygdala. Finally, improvement of psychomotor retardation was positively correlated with an increase of within-posterior DMN connectivity.

**Conclusions:**

We demonstrate that ECT induces a significant increase of connectivity at both the whole brain and within-network level. Furthermore, we provide first evidence on the association between an increase of within posterior DMN connectivity and an improvement of psychomotor retardation, a core symptom of depression.

**Disclosure:**

No significant relationships.

